# Erythropoietin and renal protection

**DOI:** 10.1186/2008-2231-21-78

**Published:** 2013-12-20

**Authors:** Azar Baradaran, Hamid Nasri, Mahmoud Rafieian-Kopaei

**Affiliations:** 1 Department of Pathology, Isfahan University of Medical Sciences, Isfahan, Iran; 2 Department of Nephrology, Division of Nephropathology, Isfahan University of Medical Sciences, Isfahan, Iran; 3 Medical Plants Research Center, Shahrekord University of Medical Sciences, Shahrekord, Iran

## Dear Editor-in-Chief

Recently much attention has been directed toward kidney protective property of erythropoietin (EPO) beyond stimulating erythropoiesis. In the study conducted by Moieni et al. the protective inpact of recombinant human erythropoietin in kidney and lung injury following renal bilateral ischemia-reperfusion (I/R) in rat model was investigated. They studied the role of EPO on renal function makers and tissue damage as well as the lung endothelial permeability in bilateral renal ischemia/reperfusion (I/R) injury model in rats [1]. In this study they found that EPO protected the kidney against I/R injury [1]. Likewise to this study, Ardalan et al. observed that EPO pretreatment could also be effective in reducing kidney and lung injury following kidney I/R and could improve the cellular antioxidant defense system in rat model. They concluded that EPO pretreatment might be effective in attenuating renal and lung injuries after renal I/R induced injury during surgical procedures, hypotension, renal transplantation and other conditions inducing renal I/R [2]. Similarly in a study on 40 male Wistar rats, conducted to test the protective effect of EPO on tubular cells, we observed that EPO was able to attenuate an increase in serum creatinine and blood urea nitrogen levels against gentamicin nephrotoxicity. Moreover, co-administration of gentamicin and EPO effectively reduced renal tissue damage induced by gentamicin, compared to the control group [3]. Our study disclosed the renal protective effect of EPO, when the drug was administered in combination with gentamicin [3-6]. Furthermore, the ameliorative property of EPO was apparent even when the drug was given after induction of kidney tubular damage by gentamicin, and it was still applicable after tissue injury [2-6]. Ameliorative effect of EPO against cisplatin nephrotoxicity was shown in the study of Kong et al. They observed that injection of EPO enhanced recovery from cisplatin-induced acute renal failure in rats through ameliorating kidney functional impairment and exerting important anti-apoptotic properties [7]. Importantly, Rjiba-Touati et al. showed that EPO administration especially in pretreatment situation protected rats against cisplatin-induced renal oxidative stress and nephrotoxicity [8]. Similarly, the renoprotective effect of cisplatin-induced kidney damage was shown in our previous study, too [9]. This indicates that EPO may have curative impact, along with its preventive property [5-9]. Hence, EPO is a promising kidney protective medication that can prevent, ameliorate, or attenuate renal tubular damage induced by gentamicin or other injurious insults such as I/R [10-13]. Previous researches also showed the efficacy of EPO on renal allograft survival, too [14-19]. In an experimental study on six-week-old male rats, treated with cyclosporine, Abe et al. found that carbamylated-EPO suppressed macrophage infiltration, phenotypic alteration of interstitial myofibroblasts and interstitial fibrosis in the cyclosporine nephropathy model. They also found that, carbamylated-EPO administration was able to decrease TGF-β1 mRNA level in cyclosporine -treated kidney. In this study, tubular apoptosis was persistently stimulated after cyclosporine treatment, while carbamylated erythropoietin significantly inhibited tubular apoptosis. They concluded that carbamylated-EPO administration was able to reduce cyclosporine -induced tubule-interstitial injury in two ways by protection of renal tubular epithelial cells from apoptosis and inhibition of interstitial fibrosis [20]. Principally, EPO triggers red blood cell maturation in bone marrow and heightens erythropoiesis [17,19-23]. It is a glycoprotein and a member of class I cytokines [1,17-19,24]. In fact renal fibrosis is the final common event in all chronic kidney disease (CKD) types with different etiologies. Persistent inflammation and transition of pericytes to myofibroblasts cause renal fibrosis and diminishing of erythropoietin production [17,19-23]. Recently, also some investigators envisage administering the EPO therapy in chronic kidney disease (CKD) prior to anemia, which will benefit renal protective effectiveness of EPO in CKD [20]. More recent findings have revealed the cellular mechanism of kidney erythropoietin synthesis and the following events leading to renal fibrosis [19,22,23,25]. Remarkably, fibroblasts from injured renal tubular epithelial cells have no significant contribution in kidney fibrosis. However kidney EPO-producing cells, originating from neural crests, differentiate into myofibroblasts after a long time exposure to inflammation. It looks that they are involved in renal fibrosis [19,22-26]. Macrophages and myofibroblasts are dominant cells causing renal fibrosis. Macrophages can be differentiated to phenotype M1 (classically activated) or M2 (wound healing) regarding to the distinctive cytokine production [19,22-26]. While, EPO can disconnect macrophages by diminishing the activity of NF-κB*, in vivo.* Thus, macrophage regulation could be one of the mechanisms that explain the anti-fibrotic effect of EPO in CKD [19,22-26]. This may explain the missing link in CKD between renal fibrosis and anemia [19,22-24]. Some recent studies have indicated the improvement of kidney function in CKD following administration of EPO [19,22-26]. Thus it may be reasonable to start erythropoietin prior to erythropoiesis in CKD, too. Hence, to better understand the renoprotective property of EPO, more experimental or clinical studies are suggested.

## Competing interests

The authors declare that they have no competing interests.
